# Prostate-Specific Antigen Trends Predict the Probability of Prostate Cancer in a Very Large U.S. Veterans Affairs Cohort

**DOI:** 10.3389/fonc.2018.00296

**Published:** 2018-08-06

**Authors:** R. Jeffrey Karnes, F. Roy MacKintosh, Christopher H. Morrell, Lori Rawson, Preston C. Sprenkle, Michael W. Kattan, Michele Colicchia, Thomas B. Neville

**Affiliations:** ^1^Mayo Clinic, Rochester, MN, United States; ^2^VA Sierra Nevada Health Care System, Reno, NV, United States; ^3^Mathematics and Statistics Department, Loyola University Maryland, Baltimore, MD, United States; ^4^VA Connecticut Healthcare System, Yale School of Medicine, New Haven, CT, United States; ^5^Department of Quantitative Health Sciences, Cleveland Clinic, Cleveland, OH, United States; ^6^Urology, University of Padua, Padua, Italy; ^7^Soar BioDynamics, Inc., Incline Village, NV, United States

**Keywords:** prostate cancer, prostate-specific antigen, PSA, PSA trend, screening, prostate cancer screening, prostate cancer biopsy, prostate cancer diagnosis

## Abstract

If prostate-specific antigen (PSA) trends help identify elevated prostate cancer (PCa) risk, they might provide early warning of progressing cancer for further evaluation and justify annual testing. Our objective was to determine whether PSA trends predict PCa likelihood. A biopsy cohort of 361,657 men was obtained from a Veterans Affairs database (1999–2012). PSA trends were estimated for the 310,458 men with at least 2 PSA tests prior to biopsy. Cancer tumors may grow exponentially with cells doubling periodically. We hypothesized that PSA from prostate cancer grows exponentially above a no cancer baseline. We estimated PSA trends on that basis along with five descriptive variables: last PSA before biopsy, growth rate in PSA from cancer above a baseline, PSA variability around the trend, number of PSA tests, and time span of tests. PSA variability is a new variable that measures percentage deviations of PSA tests from estimated trends with 0% variability for a smoothly increasing trend. Logistic regression models were used to estimate relationships between the probability of PCa at biopsy and the trend variables and age. All five PSA trend variables and age were significant predictors of prostate cancer at biopsy (*p* < 0.0001). An overall logistic regression model achieved an AUC of 0.67 for men with at least 4 tests over at least 3 years, which was a substantial improvement over a single PSA (AUC 0.58). High probability of PCa was associated with low PSA variability (smooth trends), high PSA, high growth rate, many tests over a long time-span and older age. For example, at 4.0 PSA the probability of cancer is 32% for 1 PSA test and increases to 68% for 8 tests over 7 years with smooth, fast growth (0% variability and 50% exponential growth). Our results show that smooth, fast exponential growth in PSA above a baseline predicts an increased probability of PCa. The probability increases as smooth (low variability) trends are observed for more tests over a longer time span, which makes annual testing worth considering. Worrisome PSA trends might be used to trigger further evaluation and continued monitoring of the trends—even at low PSA levels.

## Introduction

Screening for prostate cancer (PCa) using the prostate-specific antigen (PSA) blood test is controversial. There is evidence that PSA screening reduces prostate cancer deaths when a low PSA threshold, such as 3.0, is used to trigger a biopsy (1–3). However, the harms from biopsy, possible diagnosis and treatment, and the risks of side effects may outweigh the lifesaving benefits for many men. The United States Preventative Services Task Force (USPSTF) has been skeptical of the net benefits of PSA screening for men of all ages and recently revised their draft recommendation for men ages 55–69 years, suggesting “individualized decision-making after discussion with a clinician, so that each man has an opportunity to understand the potential benefits and harms of screening and to incorporate his values and preferences into his decision” (C recommendation with D recommendation against PSA-based screening for men age 70 years and older) (4).

New screening technologies and treatment methods may reduce the harms and make screening more appealing. Increased use of active surveillance (5) and treatments with reduced side effects including investigational focal therapy (6) may reduce the harms associated with diagnosis. New panels of blood tests (7–9), ultrasound imaging (10), and multi-parametric MR imaging (11) appear to offer substantial improvements over PSA screening in terms of identifying high risk prostate cancers. Online risk calculators (12, 13) consider more variables than PSA and allow personal risk assessment. They may provide men and their physicians with enough confidence to delay or avoid biopsies that might be premature using only PSA and a low threshold. Delay or avoidance of premature biopsies would reduce potential harms and make screening more appealing.

Some of the new screening technologies are expensive and probably not cost effective for initial screening of a large population. Although PSA is not a cancer-specific marker, it is a low-cost predictor of elevated cancer risk that can trigger the use of other more effective screening methods rather than trigger a biopsy with potential harms. Therefore, PSA is likely to remain the first step in an improved new screening paradigm until a better low-cost alternative is developed. Possible continued use of PSA testing raises the question of whether analysis of a series of PSA tests might provide more information than a single PSA test.

We are not the first group to ask this question. Over two-decades ago, some progressing cancers were shown to produce exponential growth in PSA (14). In response, the annual rate of change in PSA, or PSA velocity (PSAV), was proposed and analyzed (15). However, many studies found that PSAV added little or no predictive value to the level of PSA alone (16–21). The concept of PSAV risk count was introduced to increase the value of PSAV by considering consecutive increases in PSA with multiple PSAVs above a threshold (22). Risk count analysis suggested that there may be information in PSA variability (23–26). Recently, a few researchers have resumed studying the exponential growth in PSA from progressing cancers, including high-grade cancer (27, 28).

We speculated that if PSA trends could help identify elevated PCa risk, they might provide early warning of progressing cancer for further evaluation and might justify annual testing. Our objective was to determine whether PSA trends predict PCa likelihood.

## Materials and methods

### Study population

We identified 361,657 PSA-tested men in United States Veterans Affairs (VA) system who underwent prostate biopsy with results collected in VA databases from 1999 to 2012. PSA trends were estimated for the 310,458 men with at least 2 PSA tests prior to biopsy. See Table 1 for a summary of men with at least 2 PSA tests for three categories (overall, diagnosed, and not diagnosed) for ranges of each of the six predictive variables described below. The protocol was approved by the University of Nevada, Reno Institutional Review Board. Informed consent was not required for the de-identified historical data that we studied.

**Table 1:** Distribution of men with at least 2 PSA tests for total, age and five PSA trend variables and for all men and those diagnosed or not diagnosed with prostate cancer.

**Distribution for Men with at Least 2 PSA Tests**						
	**All Men Biopsied**		**Cancer Diagnosed**		**Not Diagnosed**	
	**Number**	**% Biopsied**	**Number**	**% Cancer**	**Number**	**% Not Ca**
**Total**	310,458	100	122,871	100	187,587	100
**AGE**						
50–59	63,708	21	23,889	19	39,819	21
60–69	155,429	50	58,715	48	96,714	52
70–79	76,526	25	32,952	27	43,574	23
**PSA**						
0–2.9	35,616	11	7,334	6	28,282	15
3–4.9	77,115	25	27,946	23	49,169	26
5–11.9	149,856	48	61,656	50	88,200	47
12+	47,871	15	25,935	21	21,936	12
**PSAvar**						
0–9.9%	140,507	45	62,448	51	78,059	42
10–19.9%	82,583	27	33,195	27	49,388	26
20–39.9%	43,727	14	14,800	12	28,927	15
40%+	43,641	14	12,428	10	31,213	17
**PSAgr**						
0–19.9%	123,193	40	41,697	34	81,496	43
20–39.9%	78,857	25	36,146	29	42,711	23
40–69.9%	47,549	15	22,305	18	25,244	13
70%+	60,859	20	22,723	18	38,136	20
**SPAN (YEARS)**						
0–1.49	63,766	21	26,907	22	36,859	20
1.5–3.49	79,777	26	29,563	24	50,214	27
3.5–7.99	82,425	27	31,531	26	50,894	27
8+	84,490	27	34,870	28	49,620	26
**TESTS**						
2–3	101,874	33	41,380	34	60,494	32
4–5	37,697	12	14,801	12	22,896	12
6–7	81,310	26	32,110	26	49,200	26
8+	89,577	29	34,580	28	54,997	29

The outcome variable was detection of PCa by prostate biopsy. In the VA corporate data warehouse (CDW), men who underwent prostate biopsy were identified by a code. Men diagnosed with PCa were identified by (1) a diagnosis code when available or (2) a surgery code in the medical record. PSA values were measured in ng/mL and identified by the presence of a Logical Observation Identifiers Names and Codes (LOINC) code 2857-1 in the CDW Patient Laboratory Chemistry (LabChem) table. Numeric values were collected when reported; but when not available, the text was parsed and analyzed to identify a PSA result. Age at biopsy was obtained from the VA vital status mini table of the CDW.

### PSA trend equations

We used a relatively simple model of PSA trends that reflects contributions from both progressing prostate cancer and the rest of the prostate that is not cancerous.

Studies of men without PCa showed very slow growth in PSA from relatively low levels for typical men (14, 28, 29). For simplicity, we chose a constant PSA level to model a no-cancer baseline (PSAn) for the rest of the prostate that is not cancerous.

Cancers may grow exponentially with cells doubling periodically. Moreover, there is some evidence that PSA from prostate cancer tends to grow exponentially (14, 27, 28). Therefore, we hypothesized that PSA from prostate cancer grows exponentially above a no cancer baseline and modeled increasing PSA from cancer, PSAc(t), using the equation:

$$\begin{array}{l} PSAc\left(t\right)=PSAc\left(t0\right)\;*\;EXP\;\left(PSAgr\;*\;\left(t-t0\right)\right) \end{array}$$

Where, PSAc(*t*0) is estimated PSA from cancer at the time of the last test (*t*0) growing at the annual exponential rate of PSAgr.

The overall PSA trend equation, PSA(t), is the sum of the no cancer baseline PSA and estimated PSA from cancer:

$$\begin{array}{lll} PSA\left(t\right) & = & PSAn+PSAc\;\left(t\right)\\ PSA\left(t\right) & = & PSAn+PSAc\left(t0\right)\;*\;EXP\;\left(PSAgr\;*\;\left(t-t0\right)\right) \end{array}$$

**Figure 1:**
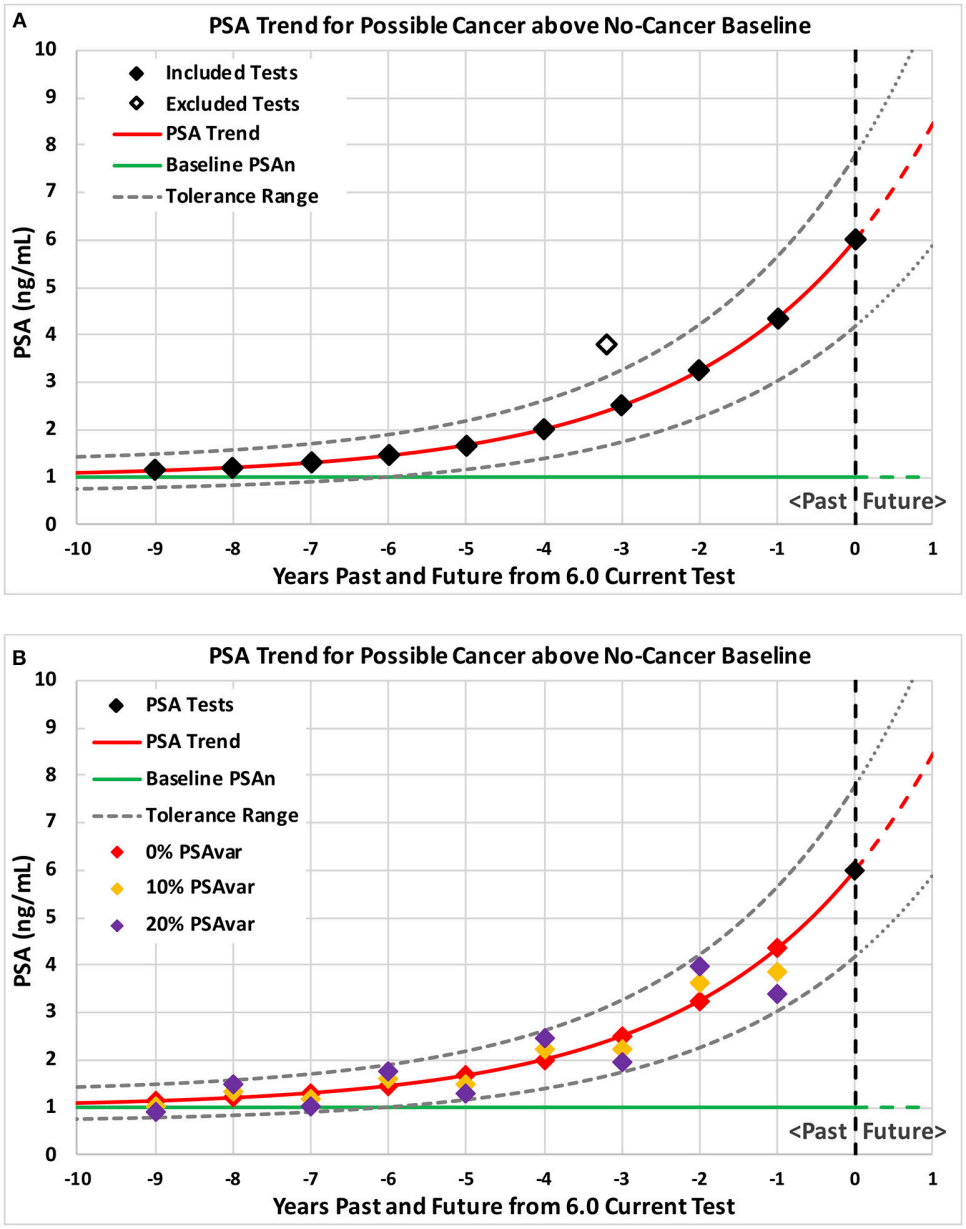
Example PSA tests and corresponding PSA trends for possible cancer above a no-cancer baseline. Example patterns of PSA tests are plotted with levels on the vertical axis and years past through current on the horizontal axis. Consistent PSA trends are shown in red above a no-cancer baseline shown in green with a tolerance range shown by dashed curves. Ten annual tests with no variation from trend plus a possible excluded high PSA test are shown on graph **(A)** followed by three sets of ten annual tests shown on graph **(B)** with 0% (red), 10% (orange), and 20% (purple) test variability around the trend (PSAvar).

Using this constant plus exponential formula, PSA trends through the last PSA test were estimated using minimum least-squared error methods. In order to avoid unreasonably low values of the no-cancer baseline (PSAn), low values were constrained to the lower of 0.8 PSA and 80% of the lowest PSA test. Figure 1A shows a PSA trend in red starting 9 years in the past and projected 1 year into the future with a no-cancer baseline of 1.0 shown as a green line.

**Figure 2:**
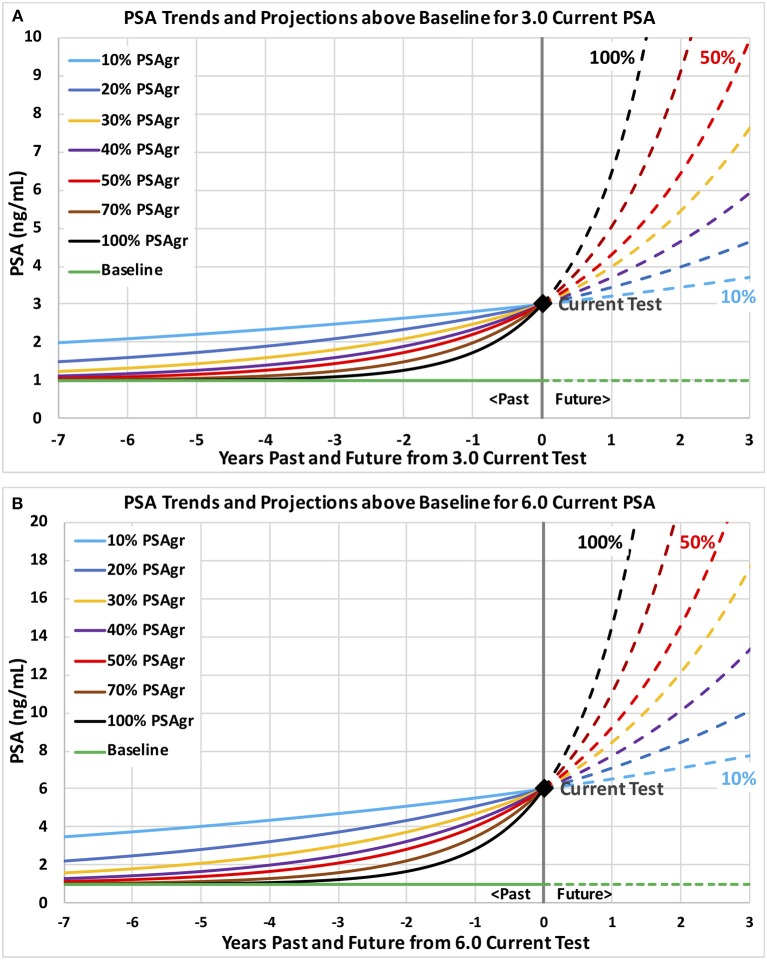
Example PSA trends above a no-cancer baseline through current PSA tests of 3.0 and 6.0 with a range of growth rates. Example PSA trends are plotted and projected with levels on the vertical axis and years past and future on the horizontal axis. Trends are shown on graph **(A)** through a current PSA test 3.0 and on graph **(B)** through 6.0. Growth rates in estimated PSA from cancer (PSAgr) range from slow (10%) shown in light blue to very fast (100%) shown in black.

In order to help visualize the implications of different values of trend variables, we calculated a range of PSA trends and presented them in Figures 2A,B.

PSA velocity (PSAV) is often defined as the annual rate of change in PSA. For our trend equation, PSAV at any point in time is defined as the slope of the PSA trend:

$$\begin{array}{l} PSAV\left(t\right)=\;PSAgr\;*\;PSAc\;\left(t\right) \end{array}$$

### Consistent PSA trend methods that exclude past high PSA tests

Temporary prostate conditions, such as prostatitis due to infection, can cause PSA to increase and then drop back to a lower underlying trend. We hypothesized that progressing prostate cancer would produce relatively steady increases in PSA unless affected by temporary conditions. Therefore, we hypothesized that increasing PSA from cancer would fall within a tolerance range of an underlying trend. To implement this hypothesis, consistent underlying trends included only tests within a tolerance range of +/−30% from the trend. High past tests above the tolerance range were iteratively excluded until all included tests were within range of the underlying consistent trend. Figure 1A shows a smooth underlying consistent trend with one excluded high past test (hollow diamond). Using the iterative process, a trend was fitted to all the PSA tests in Figure 1A. The high past test (hollow diamond) was identified as above the tolerance range and excluded for the next iteration. In the next iteration, a trend was fitted to the remaining included tests (solid black diamonds). All remaining tests were within the new tolerance range (dashed black curves) and the underlying trend (red curve) was accepted as consistent with one past high test excluded.

### PSA trend variables

For each man with two or more PSA tests prior to biopsy, we estimated consistent underlying PSA trends using the methods described above and determined values for five descriptive variables: last PSA before biopsy (PSA), growth rate in PSA from cancer above a baseline (PSAgr), PSA variability around the trend (PSAvar), number of PSA tests (Tests), and time span of the tests (Span). The data strength of each trend was described by the number of PSA tests (Tests) and time span of the tests (Span), where Span is the time (in years) between the first and last PSA test prior to biopsy. More tests over longer time produce stronger trends and, presumably, more valuable information.

We hypothesized that unsteady (variable) increases in PSA might indicate temporary conditions rather than progressing prostate cancer. We characterized unsteady increases by PSA test variation around the consistent underlying PSA trend. PSA variation (PSAvar) was calculated as the average absolute percentage deviation from the consistent underlying trend for all PSA test values, including any excluded past high tests. Figure 1B shows three different sets of tests that produce the same underlying trend but with different variability (PSAvar). A smooth trend with 0% variability is shown by the red diamonds that fall on the red trend. A low variability trend with 10% PSAvar is shown by the orange diamonds that fall slightly above and below the red trend. A modestly variable trend with 20% PSAvar is shown by the purple diamonds that fall above and below the red trend near the tolerance range (dashed black curves).

### Statistical analyses of PSA trend results

Logistic regression models were used to estimate relationships between the probability of PCa at biopsy and the trend variables and age. The 6 variables considered were: Age, PSA, PSAvar, PSAgr, Tests(number), and Span(years). Models were developed using all appropriate data and for men with stronger trends with at least 4 PSA tests over at least 3 years. When only one PSA value was available before biopsy, only PSA and Age were considered for separate models with the results used for reference.

For men with PSA trends estimated, logistic regression models were developed in three steps and fitted using R (version 3.3.1) to combinations of variables including transformations (natural log and natural log squared) for all steps and interactions for Steps 2 and 3, as described below. Areas (AUCs) under the receiver operator characteristic (ROC) curves were calculated for all models using all the data. In addition, the data was repeatedly divided into training and testing sets. Models were fit using the training data and AUCs were calculated for those models applied to the corresponding testing sets.

Step 1: We considered the following variables and their transforms:

- Age and Age^∧^2
- ln(PSA+1) and ln(PSA+1)^∧^2
- ln(PSAgr+1) and ln(PSAgr+1)^∧^2
- ln(PSAvar+1) and ln(PSAvar+1)^∧^2
- ln(Tests+1) and ln(Tests+1)^∧^2
- ln(Span+1) and ln(Span+1)^∧^2

Natural logs (ln) were used to allow for diminishing returns in the trend variables with the value of 1 added to each of those variables in the conventional way to consider 0 values. Squared terms were used to allow flexibility in the shape of the overall impact of each variable. See Table 2 for the model coefficients.

**Table 2:** Logistic regression model for Step 1: variables, estimated coefficients, z values, and *p* values.

**Variable**	**Coefficient**	**z value**	**p value**
Intercept	1.280e+00	3.841	0.000122
Age	−1.364e−01	−13.700	< 2e−16
ln(PSA+1)	6.761e−01	29.162	< 2e−16
ln(PSAgr+1)	1.887e+00	46.842	< 2e−16
ln(PSAvar+1)	−2.821e+00	−40.353	< 2e−16
ln(Tests+1)	1.863e+00	19.416	< 2e−16
ln(Span+1)	−7.218e−01	−26.126	< 2e−16
Age^∧^2	1.085e−03	14.329	< 2e−16
ln(PSA+1)^∧^2	−2.407e−02	−4.965	6.88e−07
ln(PSAgr+1)^∧^2	−1.621e+00	−47.606	< 2e−16
ln(PSAvar+1)^∧^2	1.877e+00	24.369	< 2e−16
ln(Tests+1)^∧^2	−5.271e−01	−20.137	< 2e−16
ln(Span+1)^∧^2	3.231e−01	34.133	< 2e−16

Step 2: We started with the variables used in Step 1 and then considered two and three variable interaction terms. To overcome the potential complexity of the models, we used the implementation of LASSO (least absolute shrinkage and selection operator) machine learning available in R in glmnet (version 2.0-5). Lasso selected the best fitting model while limiting complexity and over-fitting.

**Figure 3:**
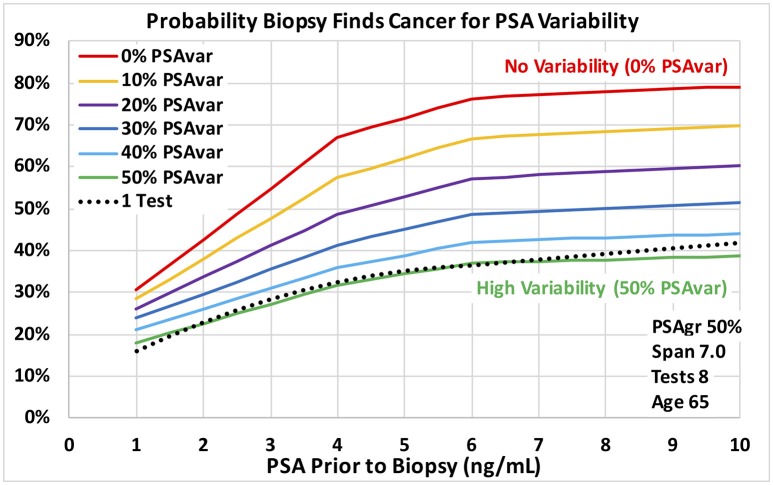
Probability a biopsy finds cancer vs. PSA before biopsy for a range of PSA test variability around PSA trend for age 65 men. Dotted reference curve reflects men with only 1 PSA test. Solid curves are based on 8 PSA tests over 7 years with moderately fast exponential growth in estimated PSA from cancer (50% PSAgr). PSA test variability around the PSA trend ranges from no variability for a smooth trend (0% PSAvar) shown in red to high variability (50% PSAvar) shown in green.

**Figure 4:**
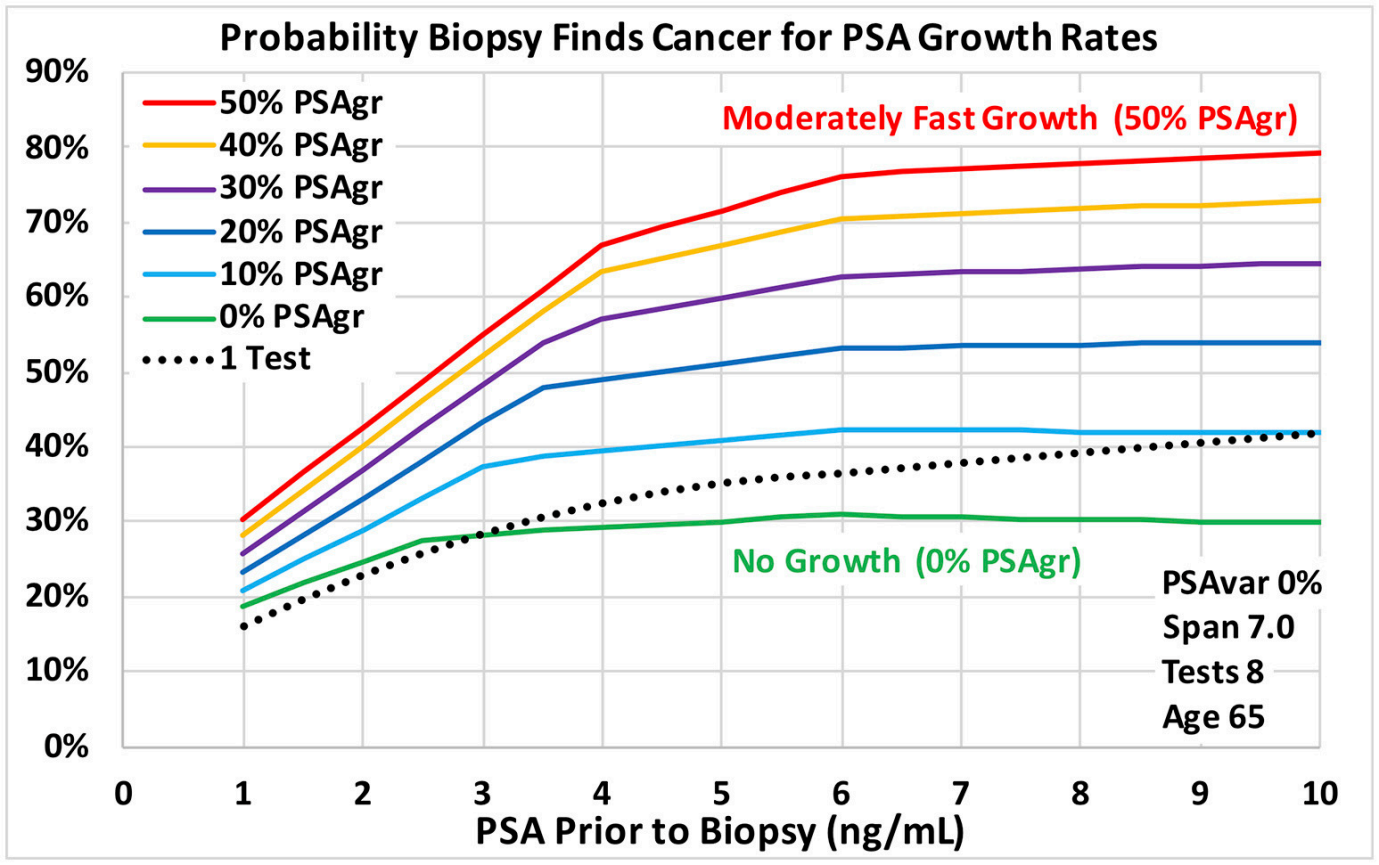
Probability a biopsy finds cancer vs. PSA before biopsy for a range of estimated growth in PSA from cancer for age 65 men. Dotted reference curve reflects men with only 1 PSA test. Solid curves are based on 8 PSA tests over 7 years with no PSA test variability for a smooth trend (0% PSAvar). Estimated exponential growth above a no-cancer baseline ranges from moderately fast (50% PSAgr) shown in red to no growth (0% PSAgr) shown in green.

**Figure 5:**
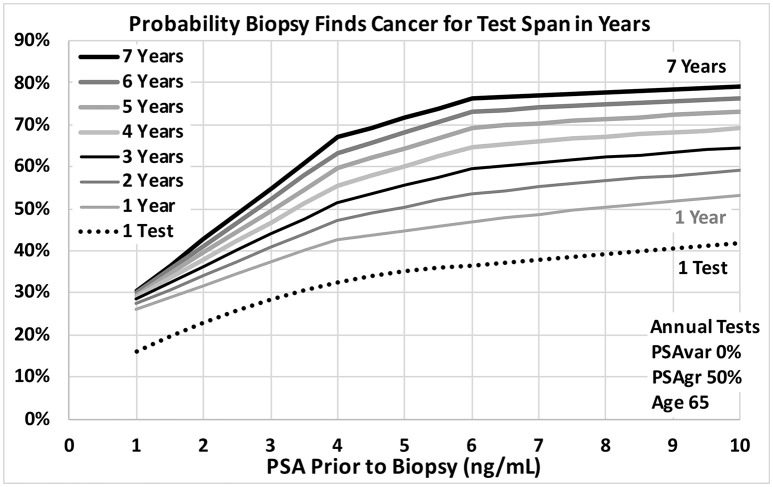
Probability a biopsy finds cancer vs. PSA before biopsy for a range of PSA test spans in years for age 65 men. Dotted reference curve reflects men with only 1 PSA test. Solid curves are based on annual PSA tests with no variability for a smooth trend (0% PSAvar) with moderately fast exponential growth in estimated PSA from cancer (50% PSAgr). Test spans range from 7 years shown in black at the top to 1 year shown in light gray.

Step 3: Analysis of simple averages for PSA ranges revealed a “steep” increase in probability up to PSA 3 or 4 followed by a “flat” increase above PSA 5 or 6. The functional forms in Step 2 could not fully model this abrupt change in shape in probability as a function of PSA. To deal with the “steep” then “flat” function of PSA for predictive purposes, we used LASSO to fit four six-variable “regional” logistic regression models for PSA ranges 0-3, 3-5, 5-12, and >12 while considering the variables used in Step 2 for consistency among the four models. We then used piece-wise linear equations to connect the models over the range of PSA values. Finally, we identified diminishing returns for PSAgr and PSAvar greater than 70% that were greater than available from the shape of the natural log transformations. Therefore, we excluded those higher values of PSAgr and PSAvar from model estimation to better predict the more prevalent and interesting lower ranges. The results of Step 3 are shown in Figures 3–**5**.

### Statistical analyses of single PSA results

Using the methods of Step 2, we developed a two-variable logistic regression model for men with only one PSA test using PSA and Age as the variables. AUC was calculated for reference.

Using the methods of Step 3, we developed four two-variable “regional” logistic regression models for men with only one PSA test using PSA and Age as the variables. The results were used as a reference for the results of Step 3 shown in Figure 3–**5**.

## Results

Table 1 summarizes the descriptive statistics for men with at least 2 PSA tests for three categories (overall, diagnosed and not diagnosed) for ranges of each of the six predictive variables. Note that over half the men have 6 or more PSA tests, and over half have a test Span of 3.5 years or more. Table 2 shows the structure and coefficients of the logistic regression model for Step 1 with no interaction terms. For reference, Figure 2A shows a 3.0 PSA current test with trends growing at rates from 10 to 100% PSAgr that are projected three years; and Figure 2B shows similar trends for a 6.0 PSA current test.

### Screening effectiveness and variable significance

For Step 1 models with no interaction terms, Age plus all five transformed trend variables and their squares were highly significant (*p* < 0.0001), as shown in Table 2. AUC was 0.65 for the model for at least 4 tests over at least 3 years and 0.64 for all Tests and Spans. For Step 2 models with interaction terms, one or more forms of all six variables were highly significant (*p* < 0.0001). AUC was 0.67 for the model for at least 4 tests over at least 3 years and 0.66 for all data. Average AUC's on test data for models estimated for separate training data decreased negligibly to 0.66 from 0.67 and to 0.65 from 0.66 respectively. For step 3 “regional” models with interaction terms, one or more forms of all six variables were highly significant (*p* < 0.0001).

For men with only one PSA test, one or more forms of both PSA and Age were highly significant (*p* < 0.0001) in the logistic regression model with an AUC of only 0.58 compared to the highest AUC for PSA trend models of 0.67.

### Predicted probabilities of prostate cancer found by biopsy

Predictions of all models were stable over many training data sets, as we expected for the very large amount of data. The probabilities of prostate cancer found by biopsy presented in this section and on Figures 3–**5** were predicted using the “regional” logistic regression models from Step 3 for PSA trends and the corresponding step for a single PSA. They show the predicted probabilities of a positive biopsy with PSA on the horizontal axis and probability on the vertical. For reference, the dotted black curves show the probability of positive biopsy using one PSA test, which is 32% at 4.0 PSA for men age 65. To visualize trends produced by a variety of trend variables, please see Figures 1A,B, 2A,B. Figures 1A,B show annual testing with 10 PSA tests over a time Span of 9 years. Figure 1A shows one additional excluded high test. Figure 1B shows example test patterns for three levels of variability: Smooth trend with 0% variability (red diamonds); Low variability trend with 10% PSAvar (orange diamonds); and Modestly variable trend with 20% PSAvar (purple diamonds). Figure 2A shows PSA trends with a range of growth rates from PSAgr 10 to 100% through a current PSA of 3.0. Figure 2B shows PSA trends with the same growth rates through a current PSA of 6.0.

Figures 3, 4 show probabilities of prostate cancer found by biopsy for strong trends with 8 tests over 7 years for age 65 men. Figure 3 assumes moderately-fast growth rate in estimated PSA from PCa (50% PSAgr). Probabilities are shown for a range of variabilities from highly variable in green (50% PSAvar) to smooth in red (0% PSAvar), which produces the highest probabilities. Figure 4 assumes smooth trends with no variability (0% PSAvar). Probabilities are shown for a range of growth rates from no growth in green (0% PSAgr) to moderately-fast growth in red (50% PSAgr), which produces the highest probabilities. Figure 5 assumes annual testing for trends that are both smooth with no variability (0% PSAvar) and moderately-fast growing (50% PSAgr) for age 65 men. Probabilities are shown for a range of test Spans from 1-year (the lowest lightest gray curve) to 7-years (top black curve), which produces the highest probabilities.

## Discussion

Using the VA's large multi-center cohort of men with PSA history who underwent prostate biopsy, we demonstrated that PSA trend variables predict the probability of PCa diagnosis at biopsy. For the first time, transforms of four PSA trend variables were shown to be statistically significant predictors of PCa diagnosis (*p* < 0.0001) when last PSA before biopsy and Age were also considered. The four PSA trend variables were: growth rate in PSA from cancer above a baseline (PSAgr), PSA variability around the trend (PSAvar), number of PSA tests (Tests) and time span of the tests (Span). The overall logistic regression model achieved an AUC of 0.67 for men with at least 4 tests over at least 3 years, which was a substantial improvement over a single PSA (AUC 0.58). PSA variability around the trend (PSAvar) is a new variable that was strongly predictive. Consider men with 4.0 PSA and 8 tests over 7 years. For moderately fast-growing trends (50% PSAgr), the probability of cancer is 32% for high PSA variability (50% PSAvar) and 68% for smooth trends with no variability (0% PSAvar). Moreover, growth rate matters most for smooth trends. Again, consider men with 4.0 PSA and 8 tests over 7 years. For a smooth trend with no variability (0% PSAvar), the probability of cancer is 30% for no growth (0% PSAgr) and 68% for moderately fast growth (50% PSAgr).

### Should PSA trends be considered in addition to PSA level for screened men?

In the evolving prostate cancer screening paradigm using new technologies, PSA is the low-cost way to begin screening a population of men. PSA levels help identify men at elevated risk who could be referred for further evaluation. The substantial increase in AUCs using PSA trends suggests that they are a low-cost way to increase initial screening effectiveness beyond PSA level alone and should be considered. The predicted results show the potentially large increase in risk when PSA trends are available compared to a single PSA. For example, at 4.0 PSA the probability of cancer is 32% for 1 PSA test and increases to 68% for 8 tests over 7 years with smooth, fast growth (0% PSAvar and 50% PSAgr).

### At what age should a man get his first PSA test?

A recent study has shown that a baseline PSA for men during midlife, as early as age 45, is highly predictive of PCa metastasis and death up to 30 years later (30). The results suggest that men should consider getting their first PSA test at a relatively young age to assess their risk and guide the timing of subsequent PSA tests. The results of our study also support obtaining a baseline PSA at a relatively young age in order to have a long time Span of tests when PSA might start to increase from progressing prostate cancer later in life. An early baseline with periodic testing will lead to long time Spans that increase the power of PSA trend analysis, as suggested by Figure 5. For example, a baseline test at age 45 and subsequent tests can provide a 5-year Span of tests by age 50 and a 10-year Span by age 55. Men at high risk because of family history, race or genetics might benefit from a baseline PSA test as early as age 40.

### How frequently should a man get a PSA test?

The recent study of baseline PSA suggests that men with elevated baseline PSA levels may benefit from more frequent PSA testing (30). Annual testing is a reasonable starting point for consideration because it can be conveniently scheduled as part of an annual physical. Men with lower baseline PSA levels, below 1.0 or 1.5, might undergo less frequent testing, at least until their PSA increases to higher levels (30). The results of our study show the value of periodic testing for PSA trend analysis with increasing benefit to more frequent testing. Annual PSA testing may be appropriate for men with PSA tests above the 1.0 or 1.5 levels suggested by the baseline PSA studies and may be convenient and comforting for men with lower PSA levels.

Our study identified some men with fast growing PSA, some of whom were subsequently diagnosed with prostate cancer. The danger is that their fast-growing PSA might reach unacceptably high levels where effective treatment becomes more difficult. Men with fast growing PSA are likely to benefit from more frequent PSA testing than annual in order to trigger further evaluation before PSA reaches high levels. Figures 2A,B show how quickly PSA can increase when growth rates are as high as 100%, shown by the steepest curves in black. Projections of PSA trends may help determine when to schedule the next PSA test. For example, the next PSA test might be scheduled when PSA is projected to increase by a substantial amount, such as 0.5. For a 3.0 current PSA, the black curve on Figure 2A shows that a very fast-growing PSA trend (100% PSAgr) will increase PSA by 0.5 in about 2 months, when a next PSA test might be scheduled. Men with an unexpected jump in PSA that may be due to a temporary condition such as prostatitis caused by infection might benefit from a follow-up PSA test 4 to 6 weeks later. The follow-up PSA test may determine if PSA continues to increase and might indicate progressing cancer or drops and suggests a temporary condition.

### How might PSA trends be used in clinical practice?

Our results suggest that a primary care physician or urologist may be able to identify increasingly risky PSA trends through monitoring and analysis. Men with elevated-risk could be evaluated using new blood tests and referred to a urologist for further evaluation if the risk of prostate cancer is high enough. Electronic medical records make it easier to capture and monitor periodic PSA tests, and eventually software might be used to estimate PSA trends and calculate trend variables to identify men at elevated risk.

What might a primary care physician or urologist do today without software assistance? The first step is to schedule a baseline PSA test, if not already available, followed by annual testing for men with a PSA level above 1.5 and possibly above 1.0. After each new PSA test the physician might review the man's full PSA history and assess the implied trend. As an example, consider 8 annual tests increasing to a 3.0 current test: 1.1, 1.1, 1.2, 1.3, 1.4, 1.7, 2.2, 3.0. On Figure 2A, this moderately fast growth in PSA to 3.0 (50% PSAgr) is shown by the red curve. This pattern of PSA tests should trigger a follow-up PSA test and further screening actions because the 55% probability of prostate cancer is relatively high, based on our results. For this example, note the steady increases in PSA with bigger increases each year and note the relatively large increase of 0.8 PSA from the penultimate to the last test (2.2–3.0). This is an alarming trend that should trigger further screening actions. In general, smooth (low variability) trends may suggest how fast PSA might be increasing to dangerously high levels and help guide the timing of next screening actions by physicians—with faster action indicated for faster growing trends. The level of concern should be much lower if PSA growth is slower with unsteady increases—especially if there are some decreases after increases that may be caused by temporary conditions.

## Limitations

The following limitations are acknowledged: First, the study is retrospective. Second, all patients underwent prostate biopsy with no record of the reason. Third, men with elevated PSA levels but no biopsy were not considered by this study. In some cases, urologists may not have biopsied those men because of past variability of PSA, including a recent decrease in PSA level. Therefore, the overall AUC might have been higher if men with elevated PSA but not biopsied had been included in the analysis. Fourth, no pathological information, such as Gleason score, stage and tumor percentage, was available to identify and model high-risk cancers. Fifth, no PSA calibration (e.g., Hybritech or WHO) was reported. However, since the PSA data came from the VA LabChem database, the majority of PSA tests probably were provided by VA labs which may have had infrequent, if any, changes in calibration. In spite of these limitations, the results are sufficiently strong to suggest that PSA trends can provide valuable information about the probability of PCa found by biopsy.

### Comparison to studies of PSA velocity

Many studies found that PSA velocity (PSAV) measured in a variety of ways, including use of the log of PSA, added little or no predictive value to the level of PSA alone (16–21). Our study suggests that a more complex model that may reflect biology by combining exponential growth in PSA from progressing cancer with a no-cancer baseline (for PSA produced by areas of the prostate without cancer) is needed to find value in PSA history. This model allows us to measure PSA variability around a consistent trend, which is an important variable not considered by most studies of PSAV. We found that growth rate (PSAgr) has little or no effect when variability (PSAvar) is high and only has the largest effect when variability is low. In addition, from a statistical perspective, PSAgr is more easily distinguishable from PSA than is PSAV, which can be highly correlated with PSA when PSAV increases with PSA for any PSAgr.

## Conclusions

In the evolving prostate cancer screening paradigm using new technologies, PSA is the low-cost way to begin screening a population of men. PSA trends may help identify men at elevated risk who could be referred for further evaluation, even at low to moderate PSA levels.

Using the VA's large multi-center cohort of men with PSA history who underwent prostate biopsy, we demonstrated that PSA trend variables predict the probability of PCa diagnosis at biopsy. For the first time, transforms of four PSA trend variables were shown to be statistically significant predictors of PCa diagnosis (*p* < 0.0001) when last PSA before biopsy and age were also considered. High probability of PCa was associated with: low PSA variability around the trend, high growth rate in PSA from cancer above a baseline, many PSA tests and long-time span of the tests—as well as with high PSA before biopsy and older age.

PSA trends improved screening effectiveness substantially over a single PSA (AUC 0.67 vs. 0.58).

The probability of prostate cancer increases as smooth (low variability) trends are observed for more tests over a longer time span, which makes annual testing worth considering. PSA trends that help identify elevated PCa risk might provide early warning of possible progressing cancer and trigger earlier evaluation using new technologies and continued monitoring of the trends with additional PSA tests.

In the clinic, primary care physicians and urologists might be able to use these results to some extent in their current practices. Electronic health records to capture PSA tests and new software to analyze the trends could help them use PSA trend analysis with low demands on their time with each patient.

## Restrictions apply to the datasets

The datasets for this manuscript are not publically available because of policies of the U.S. Department of Veterans Affairs. For our study, IRB approval was needed for our VA principal investigator to request that de-identified data from the VA corporate data warehouse (CDW) be placed in a secure workspace in the VA Informatics and Computing Infrastructure (VINCI) system where analysis was conducted. The de-identified data was not allowed to be removed from the secure VINCI workspace. We believe that a similar process would be required for others to obtain access to the datasets used for this manuscript.

## Author contributions

RK, FM, CM, LR, PS, MK, MC, and TN: Critical revision of the manuscript for important intellectual content, study concept and design, acquisition, analysis, or interpretation of data; RK, LR, FM, and CM: Study supervision; FM: full access to all of the data in the study and takes responsibility for the integrity of the data and the accuracy of the data analysis. RK, FM, CM, MC, PS, and TN: Drafting of the manuscript; FM, CM, MK, and TN: Statistical analysis; RK, FM, LR, and TN: Administrative, technical, or material support; All authors contributed to manuscript revision, read and approved the submitted version.

### Conflict of interest statement

TN is a prostate cancer survivor who wants to help other men make better prostate cancer screening decisions. Toward that end, he founded Soar BioDynamics, Inc., a small start-up company, that he has financed personally with his family. The remaining authors declare that the research was conducted in the absence of any commercial or financial relationships that could be construed as a potential conflict of interest.
